# Proximity to COVID-19 vaccination sites and vaccine uptake: the role of gender and vaccine distrust

**DOI:** 10.3389/fpubh.2025.1569280

**Published:** 2025-04-10

**Authors:** Ye Luo, Patricia Carbajales-Dale, Miao Li, William Haller, Yu-Bo Wang

**Affiliations:** ^1^Department of Sociology, Anthropology and Criminal Justice, Clemson University, Clemson, SC, United States; ^2^Center for Geospatial Technologies, Clemson University, Clemson, SC, United States; ^3^School of Mathematical and Statistical Sciences, Clemson University, Clemson, SC, United States

**Keywords:** COVID-19, vaccine uptake, proximity to vaccination sites, gender, vaccine distrust

## Abstract

**Objectives:**

Despite availability of vaccines proven to prevent severe illness, hospitalization, and death from COVID-19, a significant portion of the population remains hesitant to get vaccinated. This study examined the association between the proximity to vaccination sites and COVID-19 vaccine uptake and the role of gender and vaccine distrust in this relationship.

**Methods:**

We used data from the COVID-19 Exposure, Prevention, and Impact Study in Upstate South Carolina of the United States which was a cross-sectional survey conducted from March 2022 to August 2022 using address-based probability sampling for a mail-to-web survey. The analysis included 255 respondents (86 men and 169 women).

**Results:**

About 75% of respondents were vaccinated. Men were more likely to be vaccinated than women (84% vs. 71%). Having 1 to 9 pharmacies nearby increased vaccination odds by 4.64 times; having 10 or more increased these odds by 3.46 times (compared to no pharmacies). Each additional kilometer to the nearest pharmacy decreased vaccination odds by 8%. Women showed weaker associations between proximity to vaccination sites and vaccine uptake compared to men. Including vaccine distrust in the model rendered the interaction term of gender and proximity to vaccination sites insignificant, highlighting distrust as a dominant factor. Further analysis showed that the effect of proximity to vaccination sites on reducing COVID-19 vaccine distrust was weaker for women.

**Conclusion:**

These findings underscore the complex interplay between access, trust, and demographic factors in determining vaccine uptake. Addressing vaccine hesitancy requires a multifaceted approach. Strategies should focus on improving access, building trust through transparent communication, and tailoring interventions to demographic-specific barriers.

## 1 Introduction

The COVID-19 pandemic caused devastating global health, economic, and societal impacts, with nearly 777 million confirmed cases and more than 7.1 million deaths worldwide by October 2024 (1). Despite the availability of vaccines proven to prevent severe illness, hospitalization, and death from COVID-19, a significant portion of the population remains hesitant to get vaccinated (2, 3). In the United States, despite widespread vaccine availability, only 69.5% of the population completed their primary vaccination series as of May 2023 when the World Health Organization (WHO) declared an end to the global Public Health Emergency for COVID-19 (4). Even fewer individuals received booster shots. Vaccine hesitancy, to delay or even reject vaccination despite its availability, has been a significant barrier to controlling the pandemic (5). There has been extensive research on the risk factors for vaccine hesitancy and vaccine hesitancy in the U.S. is often linked to political polarization, misinformation, and concerns over side effects (6). More hesitant populations include younger adults, racial minorities, and people living in rural areas (7–11).

Research into vaccine behavior often uses models such as the Health Belief Model, which suggests that people’s decisions to vaccinate are influenced by factors like perceived susceptibility to the disease, the severity of potential illness, and perceived barriers to vaccination (12). The proximity to vaccination sites has been identified as a critical factor influencing COVID-19 vaccine uptake. Research indicates that individuals living closer to vaccination centers are more likely to receive the vaccine. For instance, research using geospatial methods in California and Chicago found that longer distances to vaccination sites were tied to reduced vaccine uptake (California) and that vaccination in an area increased once a nearby vaccination site opened (Chicago) (13). These findings align with evidence from other regions, such as, Pakistan, Iran and India, where proximity to healthcare facilities has been shown to enhance immunization rates (14).

Although the relationship between proximity to vaccination sites and vaccine uptake has been studied in the past, research on gender difference in this relationship is limited. To be sure, gender difference in COVID-19 vaccine uptake has been a significant area of research and the evidence is mixed. Several studies have found that women are less likely to accept or receive the COVID-19 vaccine compared to men (15–19). Other studies have reported no significant gender differences in COVID-19 vaccine acceptance (20–22). In the United States, while women had higher COVID-19 vaccination rates than men during the initial vaccine rollout, the difference lessened over time (23). These studies suggest that the relationship between gender and vaccine acceptance can vary depending on the specific population or setting. In addition, risk factors for vaccine hesitancy may vary by gender. Our study extends previous research by examining whether there is a gender difference in the effects of proximity to vaccination sites on vaccine uptake.

We also investigate whether distrust in the safety and effectiveness of the COVID-19 vaccine helps explain the connections between proximity to vaccination sites and vaccine uptake. Strong evidence suggests that lower vaccine uptake is linked to distrust in the COVID-19 vaccine (24–29). Additionally, research indicates that being closer to vaccination sites can boost vaccine trust by improving accessibility, fostering community engagement, and building trusted relationships with healthcare providers (30–33). However, few studies have explored whether vaccine distrust accounts for the relationship between proximity to vaccination sites and vaccine uptake.

This study focuses on distance versus proximity as one of the structural barriers to COVID-19 vaccine uptake. Our research questions are: (1) whether there is a positive relationship between proximity to COVID-19 vaccination sites and COVID-19 vaccine uptake; (2) whether the relationship between proximity to vaccination sites and vaccine uptake varies by gender; and (3) whether the relationship between proximity to vaccination sites and vaccine uptake is explained by trust in the safety and effectiveness of COVID-19 vaccines. Studying COVID-19 vaccine uptake is crucial for understanding vaccination trends, addressing barriers like hesitancy or access issues, and promoting equitable healthcare. It helps inform public health campaigns, improve vaccine distribution, and guide policies to ensure widespread coverage, reducing transmission, severe illness, and healthcare strain. By identifying disparities and tailoring interventions, such research supports vulnerable populations and enhances preparedness for future pandemics, ultimately contributing to more resilient and inclusive healthcare systems.

## 2 Methods

### 2.1 Participants

We used data from the COVID-19 Exposure, Prevention, and Impact Study in Upstate South Carolina. According to the 2020 U.S Census, the four Upstate South Carolina counties included in this study had 412,511 housing units in 2020 (Anderson: 89,123; Greenville: 226,215; Oconee: 40,788; Pickens: 56,385) and as of 2022, these counties had an estimated civilian, non-institutionalized population of 753,247 (Anderson: 161,979; Greenville: 417,463; Oconee: 64,972; Pickens: 108,833) (34). The four counties form a vital part of Upstate South Carolina which have thriving industrial sectors, with strong contributions from manufacturing, automotive, and advanced materials industries, while still maintaining expansive rural areas where agriculture and natural scenic landscapes play a vital role. In addition, these counties are a significant part of the core of the most populous region of South Carolina (the Greenville-Spartanburg, Anderson Combined Statistical Area) and not particularly unique in terms of population characteristics, economic structure, and social and political attitudes compared to the wider foothills region of the Southern Appalachian Mountains, which also includes parts of Virginia, Tennessee, North Carolina, and Georgia. According to the Appalachian Regional Commission (35), the Southern Appalachian Region in 2022 was the most populous (8,762,878) (p. 21, Table 3.1) and the fastest growing (+11.8% since July 1st, 2010; p. 8, Table 1.1) subregion of the Appalachian Region overall.

During the pandemic, both the South Carolina Department of Health and Environmental Control (DHEC) and the Centers for Disease Control and Prevention (CDC) strongly recommended that individuals aged 6 months and older receive the COVID-19 vaccine to protect themselves and others (36). However, COVID-19 vaccinations were not mandated. Employers implementing vaccine requirements must accommodate valid religious and medical exemptions, ensuring compliance with state laws designed to protect individual rights (37).

This cross-sectional survey was conducted by Clemson University. Data were collected from March 2022 to August 2022 using an address-based sampling mail-to-web survey. A random sample of 1,500 household addresses in the four counties were initially selected and the adult (age 18 or over) in the household who had the most recent birthday was asked to complete the survey. A recruitment letter was mailed to these addresses, and the respondents were instructed to complete the survey on the web. Two follow-up letters were mailed, and for those who still had not responded, a fourth mailing included a paper questionnaire for the respondent to complete and return. Upon completion of the survey, respondents received a $20 gift card via mail or email. Among 1,402 valid addresses, 302 completed the survey (213 web surveys and 89 mail surveys), with a response rate of 21.4%. Although our response rate was low, it was similar to other surveys conducted during the pandemic. For example, the US General Social Survey conducted by the National Opinion Research Center also used web and mail mixed mode survey in 2021 and had a response rate of 17.4% (38). Female (66.2%), White (87.2%), and older adults (65 and older) (32.6%) were over-represented in our sample compared to the 2020 Census Bureau’s estimates (52.1, 76.1, 23.1% respectively) (34). The study was approved by the Institutional Review Board at Clemson University and all respondents have consented to participate in the survey. After list-wise deletion of missing cases, the analytic sample included 255 respondents with 86 men and 169 women. We used spatial network analysis from the respondents’ addresses to operationalize the variables measuring the proximity to vaccination sites, including the number of pharmacies within a 10-min driving distance from the respondent’s home and the distance to the closest pharmacy.

### 2.2 Measures

#### 2.2.1 COVID-19 vaccine uptake

Respondents were asked whether they have been fully vaccinated, partially vaccinated, or have not been vaccinated for COVID-19. A dummy variable was created to indicate vaccination status with those who have been fully or partially vaccinated coded 1.

#### 2.2.2 Proximity to vaccination sites

The number of pharmacies within a 10-min driving distance from the respondent’s home and the distance of the closest pharmacy from the respondent’s home were calculated to indicate the proximity to vaccination sites. Because the number of pharmacies within 10 min was highly skewed with a range of 0 to 49, we recoded it into three categories: none, 1–9 pharmacies, and 10 or more pharmacies. This cut-point was chosen to ensure there is a sufficient number of respondents in the analysis of the interaction effect of gender and the proximity measures.

#### 2.2.3 Vaccine distrust

Respondents were asked to what extent they agree with the following statements: (i) COVID vaccine safety data is often fabricated; (ii) people are deceived about COVID-19 vaccine efficacy; (iii) vaccine efficacy data is often fabricated; and (iv) people are deceived about vaccine safety. The 5-point response options ranged from strongly disagree to strongly agree. A vaccine distrust scale was calculated by averaging respondents’ answers to the four statements. It ranged from 1 to 5 with higher values associated with greater levels of distrust (Cronbach’s alpha = 0.96).

#### 2.2.4 Demographic covariates

Sociodemographic covariates include age in years, gender, LGBTQ++ identity, race (White, Black and other races), marital status (married, divorced/separated, widowed, and never married), presence of children under 18 in household, education level (ranging from 1 “8th grade or less” to 9 “graduate/professional degree”), household income level (ranging from 1 “Less than $10,000” to 11 “$100,000 or more”), employment status, political ideology (liberal, moderate, conservative), religion (Protestant, Catholic, Other religion, and no religion), respondent’s number of chronic conditions (top-coded to 4 when there were more than 4 conditions), and whether any household member had medical conditions that increased their risk for COVID-19 infection.

### 2.3 Statistical analysis

We first calculated descriptive statistics for all respondents and then separately for men and women. T-test or Chi-square tests were used to test the significance of gender differences. We then used binary logistic regression to examine the relationship between proximity to vaccination sites and COVID-19 vaccine uptake. We estimated three models for each measure of proximity to vaccination sites, described above. The first model included one proximity measure and all sociodemographic covariates, the second model added the interaction term between gender and that proximity measure to see whether the relationship between proximity to vaccination sites and vaccine uptake varied by gender, and the third model added the vaccine distrust scale to see whether vaccine distrust explained the effect of proximity to vaccination sites and the interaction effect of gender and proximity to vaccination sites. In the next set of analysis, we examined the association between proximity to vaccination sites and vaccine distrust using ordinary least squares regression (OLS). We estimated two models for each measure of proximity. The first model included one proximity measure and all sociodemographic covariates, and the second model added the interaction term between gender and that proximity measure to see whether the relationship between the proximity measure and vaccine distrust varied by gender. In addition, we assessed the adequacy of our sample size for these analyses.

## 3 Results

### 3.1 Descriptive statistics

About 75% of respondents have been vaccinated. About 17% had no pharmacies within 10 min, 41% had 1 to 9 pharmacies, and 42% had 10 or more pharmacies. On average, the distance to the closest pharmacy was about 6 kilometers. On a scale from 1 to 5, the vaccine distrust scale had a mean of 2.87. Men were more likely to have been vaccinated than women (84% versus 71%, *p* < 0.05). In our sample, men were older, had a lower proportion with young children in the household, had higher household income, had a higher proportion being conservative and a lower proportion being moderate, and had more chronic conditions than women (Table 1).

**Table 1:** Descriptive statistics for all respondents and by gender.

Variables	All (*N* = 255)	Men (*N* = 86)	Women (*N* = 169)	P of gender difference
	Mean (std)/%	Mean (std)/%	Mean (std)/%	
Vaccinated	75.3	83.7	71.0	*
# Pharmacies within 10 min				
None	17.3	22.1	14.8	
1–9	40.8	32.6	45.0	
10+	42.0	45.3	40.2	
Distance to closest pharmacy (km)	5.87 (4.93)	6.09 (4.93)	5.75 (4.93)	
Vaccine distrust scale	2.87 (1.27)	2.84 (1.24)	2.88 (1.28)	
Age	52.89 (18.61)	57.24 (18.06)	50.69 (18.51)	**
Race				
White	87.4	88.4	87.0	
Black	5.9	2.3	7.7	
Other race	6.7	9.3	5.3	
LGBTQ++	5.9	5.8	5.9	
Marital status				
Married	68.6	73.3	66.3	
Divorced/separated	11.8	9.3	13.0	
Widowed	5.9	3.5	7.1	
Never married	13.7	14.0	13.6	
Kids under 18 in HH	32.2	22.1	37.3	*
Education level	6.73 (2.01)	6.87 (1.93)	6.66 (2.04)	
HH income level	7.07 (3.38)	7.87 (3.19)	6.66 (3.41)	**
Working	55.7	58.1	54.4	
Political ideology				**
Liberal	24.7	26.7	23.7	
Moderate	25.9	12.8	32.5	
Conservative	49.4	60.5	43.8	
Religion				
Protestant	66.7	65.1	67.5	
Catholic	11.4	11.6	11.2	
Other religion	4.3	4.7	4.1	
No religion	17.6	18.6	17.2	
# Chronic conditions before COVID	0.89 (1.11)	1.07 (1.18)	0.79 (1.07)	+
HH member having COVID risk conditions	35.7	31.4	37.9	

*T*-test was used for continuous variables and Chi-square test was used for categorical variables to test the significance of gender differences. ** *p* < 0.01, * *p* < 0.05, + *p* < 0.1 (two-tailed tests).

### 3.2 Proximity to vaccination sites and vaccine uptake

The results from binary logistic regression on vaccination status showed that proximity to vaccination sites was significantly associated with vaccine uptake controlling for sociodemographic covariates (Table 2, Model 1). Respondents who had 1–9 pharmacies located within 10 min from their home were 4.64 times (*p* < 0.01) and respondents who had 10 or more pharmacies within 10 min from their home were 3.46 times (*p* < 0.05) more likely to have been vaccinated, compared to respondents who had no pharmacy located within 10 min from their home. Living one-kilometer longer distance from the closest pharmacy was associated with 8% (*p* < 0.05) decrease in the odds of being vaccinated.

**Table 2:** Odds ratios from binary logistic regressions of measures of proximity to vaccination sites, interactions between gender and proximity measures, and vaccine distrust scale on vaccination status (Vaccinated = 1; Not vaccinated = 0).

VARIABLES	Model 1a	Model 1b	Model 2a	Mode 2b	Model 3a	Model 3b
# Pharmacies within 10 min (ref = 0)						
1–9	4.64**		13.15**		8.78*	
10+	3.46*		13.74**		13.59*	
Distance to closest pharmacy (km)		0.92*		0.82**		0.82*
Female X # Pharmacies within 10 min						
1–9			0.18		0.25	
10+			0.11*		0.24	
Female X Distance to closest pharmacy				1.18+		1.09
Vaccine distrust scale					0.14**	0.13**
Age	1.04*	1.04*	1.04*	1.04*	1.07**	1.07**
Female	0.51	0.57	2.29	0.19*	2.09	0.43
Race (ref = White)						
Black	0.61	0.57	0.71	0.66	0.41	0.35
Other race	10.98+	11.32+	11.48+	12.15+	12.61+	9.90+
LGBTQ++	8.91+	11.37+	8.58+	10.36+	3.87	3.45
Marital status (ref = Married)						
Divorced/separated	2.17	1.85	2.00	1.81	1.46	1.35
Widowed	0.70	0.72	0.67	0.74	0.42	0.54
Never married	1.07	0.97	1.25	1.12	0.56	0.59
Kids under 18 in HH	0.63	0.68	0.62	0.66	0.59	0.63
Education level	1.11	1.13	1.10	1.11	0.99	1.01
HH income level	1.19*	1.16*	1.19*	1.16*	1.12	1.13
Working	1.08	1.17	1.00	1.11	1.91	1.81
Political ideology (ref = Conservative)						
Moderate	3.49*	3.24*	3.47*	3.41*	1.01	1.02
Liberal	9.93**	9.36**	9.36**	8.94**	1.31	1.38
Religion (ref = Protestant)						
Catholic	10.29*	10.01*	10.89*	10.26*	10.27*	9.69+
Other religion	0.23	0.18+	0.21	0.16+	0.21	0.20
No religion	0.37	0.43	0.37	0.41	0.46	0.52
# Chronic conditions before COVID	0.98	0.98	0.97	0.98	1.06	1.03
HH member has COVID risk conditions	2.11	2.19+	2.22+	2.17+	1.12	1.30
Constant	0.01**	0.07*	0.01**	0.15	8.05	168.31*

*N* = 255. ** *p* < 0.01, * *p* < 0.05, + *p* < 0.1 (two-tailed tests).

When the interaction term between gender and the proximity measure was added in Model 2, the interaction term between female gender and 10 or more pharmacies was significant and indicated that the association between number of pharmacies nearby and vaccination status was weaker for women than for men, especially when they had 10 or more pharmacies nearly (OR for interaction term = 0.11, *p* < 0.05). A marginally significant interaction term between female gender and distance to the closest pharmacy indicated a weaker effect of distance to the closest pharmacy on vaccination status for women than for men.

When the vaccine distrust scale was added in Model 3, vaccine distrust had a strong negative association with vaccine uptake. With one unit increase on the distrust scale, the odds of vaccine uptake decreased by more than 85% (*p* < 0.05). It is also interesting to see that the interaction terms between gender and measures of the proximity to vaccination sites were no longer significant.

### 3.3 Proximity to vaccine sites and vaccine distrust

The results from OLS regression on vaccine distrust scale showed that controlling for sociodemographic covariates, respondents who had 1 or more pharmacies nearby had a lower level of vaccine distrust although only marginally significance was detected between those with and without 1 to 9 pharmacies nearby (Table 3, Model 1). The association between distance to closest pharmacy was positive, but not significant. When the interaction terms between gender and measures of the proximity to vaccination sites were added, the main effects of the proximity measure remained the same directions but became significant while the interaction terms were significant. The main effects indicate that for men, having 1 to 9 pharmacies within 10 min was associated with 0.6 point decrease on vaccine distrust scale (*p* < 0.1), and 10 or more pharmacies within 10 min was associated with 0.86 point decrease in vaccine distrust scale (*p* < 0.01), while 1 km longer distance to the closest pharmacy was associated 0.6 points increase in vaccine distrust scale (*p* < 0.05). For the number of pharmacies nearby, the coefficients for the interaction terms were positive indicating its effect was weaker for women than for men. For distance to the closest pharmacy, the coefficient for the interaction term was negative, also indicating a weaker effect of distance to closest pharmacy for women than for men.

**Table 3:** Unstandardized regression coefficients from OLS regressions of measures of proximity to vaccination sites and interactions between gender and proximity measures on vaccine distrust scale.

Variables	Model 1a	Model 1b	Model 2a	Model 2b
# Pharmacies within 10 min (ref = 0)				
1–9	−0.35+		−0.60+	
10+	−0.26		−0.86**	
Distance to closest pharmacy (km)		0.01		0.06*
Female X # Pharmacies within 10 min				
1–9			0.45	
10+			1.00**	
Female X Distance to closest pharmacy				−0.07*
Age	−0.00	−0.00	−0.00	−0.00
Female	0.13	0.11	−0.46	0.53*
Race (ref = White)				
Black	−0.12	−0.13	−0.19	−0.19
Other race	−0.47+	−0.51+	−0.47+	−0.53*
LGBTQ++	−0.14	−0.16	−0.08	−0.10
Marital status (ref = Married)				
Divorced/separated	−0.25	−0.23	−0.25	−0.23
Widowed	0.00	−0.00	0.01	−0.03
Never married	−0.24	−0.21	−0.31	−0.31
Kids under 18 in HH	−0.02	−0.02	−0.02	−0.01
Education level	−0.09*	−0.09*	−0.08*	−0.08*
HH income level	−0.07**	−0.06**	−0.07**	−0.07**
Working	0.36*	0.34*	0.39**	0.37*
Political ideology (ref = Conservative)				
Moderate	−0.97**	−0.96**	−1.02**	−0.99**
Liberal	−1.69**	−1.67**	−1.72**	−1.68**
Religion (ref = Protestant)				
Catholic	−0.26	−0.26	−0.27	−0.26
Other religion	0.23	0.26	0.31	0.32
No religion	0.21	0.18	0.26	0.20
# Chronic conditions before COVID	0.00	0.00	−0.00	−0.00
HH member has COVID risk conditions	−0.37*	−0.36*	−0.37*	−0.35*
Constant	4.94**	4.63**	5.32**	4.38**
R square	0.43	0.43	0.45	0.44

*N* = 255. ** *p* < 0.01, * *p* < 0.05, + *p* < 0.1 (two-tailed tests).

### 3.4 Sample size analysis

In order to assess the adequacy of our sample size, we conducted a *post hoc* power analysis using Stata. For linear regression on vaccine distrust, assuming a 5% significance level and 80% power, with 23 covariates, a sample of 255 respondents has the power of 17.6% to detect a small effect (*R* square = 0.02), 96.9% to detect a medium effect (*R* square = 0.13), and 100% to detect a large effect (*R* square = 0.26). To detect a medium effect with 23 predictors, the minimum sample size required is 167 respondents. Power analysis on the main effects and measures of proximity to vaccination sites and their interactions with gender showed that our sample has 86% power to detect 3% increase in the R square when these variables were added to the models. There is less consensus on sample size for logistic regression to obtain stable estimates in the literature. Scott (39) suggests that sample sizes of less than 100 should be avoided and that 500 observations should be adequate for almost any situation. Our power analysis for the logistic regressions on vaccine uptake showed that for the model on the number of pharmacies within 10 min driving, our sample had 60% power and for the model on distance to the closest pharmacy, our sample had over 90% power.

We also ran additional analysis to test the robustness of our findings. First, we estimated OLS regression models for the vaccine uptake variable. Second, we removed some of the variables which were not significant in the models. Both approaches produced consistent results, with the main effects of proximity to vaccination sites and the interaction effects of gender and proximity measures remaining statistically significant.

## 4 Discussion

This study examined the relationship between proximity to COVID-19 vaccination sites and COVID-19 vaccine uptake. The findings underscore the complex interplay between access, trust, and demographic factors in determining vaccine uptake as the results show that while lack of proximity to pharmacies significantly influenced vaccination rates, their effects were modulated by gender and vaccine distrust.

Consistent with the Health Belief Model and previous research, this study found that a greater number of nearby pharmacies and shorter travel distance to pharmacies were associated with higher odds of vaccine uptake. Studies have demonstrated that reducing travel distances to vaccination sites can mitigate barriers to vaccine uptake by decreasing social and physical discomforts related to accessing healthcare during the pandemic, especially for populations at higher risk of severe COVID-19 outcomes (13, 40). Findings from these studies support public health initiatives aimed at improving access to vaccination information and sites, such as on-site vaccination services, mobile vaccination units, and pop-up clinics (41, 42). These initiatives can provide vaccines in community settings, thereby reducing the need for individuals to travel long distances. Such strategies are particularly beneficial in addressing the needs of populations with limited mobility or those living in areas with few healthcare resources (42, 43).

Our finding that women were less likely to be vaccinated than men is consistent with the majority of previous studies (15–18, 23). Women’s lower vaccination rate could be attributed to several reasons, including women being more affected by COVID-19 misinformation (16), having lower risk perception of the disease (17, 44), potentially being more sceptical about vaccine safety and effectiveness (15, 45, 46), and greater caregiving responsibilities (47, 48). It should be noted that the gender difference was not statistically significant in our multivariate regression models, suggesting that other factors may have stronger and more direct effects on vaccine uptake.

Previous research suggests that proximity to vaccination sites may impact women more strongly due to caregiving responsibilities, time constraints, and limited transportation access (47–49). Women often balance work and family duties, making distant sites challenging to reach. Interestingly, contrary to such expectations, the association between proximity to vaccination sites and vaccine uptake was weaker for women than for men in our data. This finding may suggest that distance to vaccination sites alone does not seem to be a major deterrence factor in women’s vaccination decision for COVID-19. Perhaps having more children in the household and the normative childcare expectations for women help explain why distance to nearest pharmacy is less of a deterrent to women’s vaccine uptake. It is also possible that men encounter other challenges, such as inflexible work hours in male-biased industries, such as construction. Additionally, men may prioritize convenience and ease, making proximity an important factor for encouraging their vaccination (50). Our finding suggests that convenient vaccination sites near workplaces or recreational areas may be key for improving men’s access. Furthermore, our analysis shows that the association between proximity to pharmacies and vaccine distrust was weaker for women than for men which may also contribute to the weaker effects of proximity to vaccination sites on vaccine uptake for women.

Vaccine distrust emerged as a critical determinant of vaccination behavior in our data. It also helps explain some of the effects of proximity to vaccination sites. This is consistent with previous research. There is strong evidence that distrust in COVID-19 vaccine is associated with lower vaccine uptake (24–27). This distrust appears to be driven by a variety of factors, including concerns about vaccine side effects and long-term safety, lack of confidence and trust in health authorities, medical institutions, and scientific experts developing and administering the vaccines, exposure to vaccine misinformation and conspiracy theories, and perceptions of lower COVID-19 severity and personal risk. These issues are especially prominent among certain demographic groups, such as Black, Hispanic, and Indigenous populations, as well as individuals with lower socioeconomic status (28). The spread of misinformation on social media has further fueled these concerns, leading to increased skepticism and delay in vaccine uptake (29).

Proximity to vaccination sites can enhance vaccine trust through increased accessibility, community engagement, and the establishment of trusted relationships with healthcare providers. When vaccination sites are easily accessible, individuals can engage directly with healthcare professionals, fostering familiarity and confidence in the vaccination process (30). Community engagement, particularly through local influencers sharing positive vaccination experiences, can further reinforce trust (31). Additionally, proximity allows for the dissemination of accurate information, countering misinformation that may breed distrust (32). The presence of trusted healthcare providers at nearby sites can facilitate open discussions about vaccine safety, enhancing individuals’ confidence in the vaccines offered (33). Overall, when vaccination sites are integrated into the community, they create an environment conducive to building trust in vaccines and the healthcare system, ultimately promoting higher vaccine uptake.

The strong negative correlation between distrust and uptake highlights the need for targeted efforts to build trust in vaccines. Interventions should address concerns about vaccine safety and efficacy while considering gendered differences in how access barriers impact vaccination decisions. To address vaccine hesitancy, public health campaigns such as the U.S. Department of Health and Human Services’ “We Can Do This” initiative have been launched (51). These campaigns rely on behavioral science to counter misinformation, promote the benefits of vaccination, and increase access (52). However, overcoming vaccine hesitancy remains a challenge, especially in politically polarized environments and areas with high levels of misinformation (29). Successful interventions will need to focus on understanding the social, psychological, and demographic factors influencing vaccine decisions and tailor public health messages accordingly to increase vaccine uptake (7).

This study has several limitations. First, the data used in this study were cross-sectional which limits our ability to establish causal directions of the relationships. Whether some of the relationships observed in this study were due to other proxy factors, such as population density, needs further investigation in future research. Second, our study used data collected in four counties in Upstate South Carolina. Because the four counties are not particularly unique in terms of population characteristics, economic structure, and social and political attitudes compared to the wider foothills region of the Southern Appalachian Mountains, our findings may be generalized to the wider foothills region of the Southern Appalachian Mountains. However, more research using data collected from other regions is needed. Third, the measures of proximity to vaccination sites did not include health clinics without a pharmacy and mobile vaccination sites. Fourth, the sample size is small for detailed analysis of gender specific risk factors and other subgroup differences. Future research using data from large samples collected during the pandemic and from other areas is also needed to verify the gender differences reported in this study. Fifth, although we used probability sampling method, White, female and older adults were overrepresented in our final sample.

In conclusion, this study highlights the intricate relationship between proximity to COVID-19 vaccination sites, demographic factors, and vaccine distrust in shaping vaccine uptake. While closer access to pharmacies was generally associated with higher vaccination rates, the effect was nuanced by gender and trust in vaccines. Women’s vaccination behavior appeared less influenced by distance to the vaccination sites, suggesting that broader systemic factors, including misinformation, caregiving responsibilities, and scepticism, may play a larger role. In contrast, men’s vaccination decisions seemed more contingent on convenience and accessibility, underlining the importance of workplace or community-based vaccination initiatives. Additionally, the critical role of vaccine distrust underscores the need for trust-building measures, such as targeted public health campaigns and localized outreach efforts, to address misinformation and promote vaccine confidence. Tailored, multifaceted strategies that consider the interplay of physical, social, and psychological factors will be essential to improving vaccine equity and ensuring effective public health interventions.

## Data Availability

The raw data supporting the conclusions of this article will be made available by the authors, without undue reservation.
